# A Regression Model for Predicting Shape Deformation after Breast Conserving Surgery

**DOI:** 10.3390/s18010167

**Published:** 2018-01-09

**Authors:** Hooshiar Zolfagharnasab, Sílvia Bessa, Sara P. Oliveira, Pedro Faria, João F. Teixeira, Jaime S. Cardoso, Hélder P. Oliveira

**Affiliations:** 1 INESC TEC, 4200-465 Porto, Portugal; silvia.n.bessa@inesctec.pt (S.B.); sara.i.oliveira@inesctec.pt (S.P.O.); jpfteixeira.eng@gmail.com (J.F.T.); jaime.cardoso@inesctec.pt (J.S.C.); 2 Departamento de Engenharia Eletrotécnica e de Computadores, Faculdade de Engenharia, Universidade do Porto, 4200-465 Porto, Portugal; 3 Departamento de Engenharia Informática, Faculdade de Engenharia, Universidade do Porto, 4200-465 Porto, Portugal; pedrommlcfaria@gmail.com; 4 Departamento de Ciência de Computadores, Faculdade de Ciência da Universidade do Porto, 4169-007 Porto, Portugal

**Keywords:** regression model, Random Forests, breast cancer, breast conserving surgery, breast deformation, shape prediction

## Abstract

Breast cancer treatments can have a negative impact on breast aesthetics, in case when surgery is intended to intersect tumor. For many years mastectomy was the only surgical option, but more recently breast conserving surgery (BCS) has been promoted as a liable alternative to treat cancer while preserving most part of the breast. However, there is still a significant number of BCS intervened patients who are unpleasant with the result of the treatment, which leads to self-image issues and emotional overloads. Surgeons recognize the value of a tool to predict the breast shape after BCS to facilitate surgeon/patient communication and allow more educated decisions; however, no such tool is available that is suited for clinical usage. These tools could serve as a way of visually sensing the aesthetic consequences of the treatment. In this research, it is intended to propose a methodology for predict the deformation after BCS by using machine learning techniques. Nonetheless, there is no appropriate dataset containing breast data before and after surgery in order to train a learning model. Therefore, an in-house semi-synthetic dataset is proposed to fulfill the requirement of this research. Using the proposed dataset, several learning methodologies were investigated, and promising outcomes are obtained.

## 1. Introduction

Breast cancer is a widely known disease that mostly affects women around the world. With one of the highest incidence rates of female cancer, the success of treatment really depends on diagnosing the cancer in its earlier stages [1]. Treatments have progressed to have lower secondary effects, but breast cancer surgery is still a reality for most patients. For decades, Mastectomy was prescribed for almost every breast cancer case with a high rate of success for removing the tumor; however, this surgical option comprises the removal of the entire breast and it has a profound impact on the aesthetic appearance and self-confidence of women [2]. With the widespread of screening mammography, the average size of the detected tumors has decreased and breast conservative treatment may be appropriate for most of patients (50–75%) with breast cancer at early stages [3]. Breast conserving surgery (BCS) is an important part of conservative treatment, comprising the excision of the tumor plus a margin of healthy tissues to eliminate cancerous cells. With better cosmesis results, BCS is nowadays the preferred alternative to mastectomy. Yet, a treatment plan is always tailored based on both medical and personal choices. Treatment options are conditioned by the biology of the tumor, the stage of breast cancer, the patient’s health conditions and preferences [1].

Several studies have shown that the survival rate is almost the same for both mastectomy and BCS, with the benefit that the second imposes less deformation on breast and a more satisfactory aesthetic outcome can be achieved [4]. Still, despite the smaller deformation after BCS, it has been reported that up to 30% of patients are dissatisfied with their post-operative appearance [5]. Actually, the final aesthetic outcome can be affected by so many different variables, from different surgical practices and expertise, to some breast specific characteristics, such as volume and density, tumor size and location, hardening the prediction and patient/surgeon communication about surgical procedure results. Patients are usually involved in the decision process regarding their surgery, but most of the time surgeons lack the means to provide visual clues about the post-surgery results of different alternatives. Even though, this is an important step, regarding the acceptance of the final outcome, as also the contribution of breasts to the sense of femininity and beauty of most women. In fact, follow-up studies after breast cancer treatment show the harmful impact of poor aesthetic results on the psychosocial health of women, who describe loss of self-esteem [2], sexual impairment [6] and dislike towards their bodies after treatment [7]. On the other hand, physicians have been recognizing the value of support decision systems for planning BCS, to compare the outcome of different surgical options and facilitate surgeon/patient communication. The value of such systems is further supported by studies confirming that women are more willing to deal with the aesthetic results when they are included in the decision process [8]. These visual sensor tools could inform better the patient about the aesthetic consequences of the treatment and improve the feeling about all the process.

The development of a planning/simulation tool for surgery demands the creation of three dimensional (3D) models of the breast that can be deformed in a realistic fashion, to reproduce known deformations imposed by surgery; however, the creation of such models is a challenging task due to the deformable characteristics of the breast, the lack of landmarks to define its shape and the complex nature of the deformations imposed by the surgery. To the best of our knowledge, there are currently no tools, other than surgical experience and clinical judgment [9], to predict the impact of BCS on the shape and deformation of the treated breast [10]. In fact, the available solutions usually rely on generic models, are mainly targeted to plastic surgery (namely breast augmentation) and do not comprise complex deformations, such as the ones resulting from BCS. Moreover, they usually demand expensive and large equipment to scan patient’s torso and require expertise to handle those scans [11].

Still, in literature, strategies to model breast deformations are abundant and designed to different applications: estimate pose transformation [12,13,14], assist registration tasks among different radiological imaging modalities [15,16,17], model breast deformation [18,19,20], guide surgery [21,22], predict the healing process of the breast after tumor removal [23,24], among others. In particular, we highlight the work of Vavourakis et al. [24], that proposed a 3D surgical simulator to predict a patient-specific outcome after BCS. This framework predicts the breast shape after surgery taking the wound healing process into account. The simulator relies on a coupled multiscale Finite Element (FE) numerical procedure to solve two mathematical models: a biochemical model for wound healing and angiogenesis, and a biomechanical model for soft tissues and pose estimation. The first considers both wound healing biochemical process and the formation of new blood vessels, while the second predicts the breast shape as function of the breast tissues mass density and the body force vector. The final shape of the breast is then predicted as an integration of both models.

The aforementioned applications have in common the use of biomechanical models to predict deformations that, due to some inherent limitations, is not the most suitable approach to include in a tool designed to be used in the daily clinical practice. First, the computational process for most algorithms might take hours, days or even almost a week, depending on the complexity of the models. As a principle aim, to ease the patient/physician communications during their consultations, the planning tool must provide the solution in an expected short time. However, the biomechanical models demand high-end requirements which are not accessible in all clinics. Considering the common computation power of the available machines in clinics, the biomechanical models might take days to provide the required planning, which is too late to satisfy the aforesaid aim. Therefore, faster methodologies should be considered instead of faster machines. Second, most models are simple representations of the breast biomechanics, using unverified parameters, posing fidelity concerns on the predictions. Third, the characterization of individual-specific parameters to create personalized models of the breast, alongside with precise representation of loading and boundary constraints during different clinical procedures, is still an unsolved challenge [25]. The aforesaid applications are intended to provide patient-specific treatment solutions. Thus, the model parameters should be easily personalized with respect to patient and tumor characterizations during the consultations. Alternative strategies to model breast deformations encompass the fitting of parametric models [26,27,28,29,30], physical equations to describe known breast deformations [31], user-intuitive parameters to change breast shape [32,33] or dataset of known cases to simulate breast surgery outcomes [34]. These strategies model the breast with limited number of parameters and produce results in a timely manner more adequate to clinical practice, but the modelling of breast deformations has still to be improved for clinical surgery planning applications.

In this study, the use of Machine Learning (ML) regression methodologies, such as Random Forests (RF) and Gradient Boosting Regression (GBR), will be explored to learn breast deformations from exemplar data.

Although ML methodologies have not been visited as a solution for real data, not only are they capable of performing the prediction in a meaningful time, but also they need less expertise for configuration. Besides the mentioned advantages, the ability of discovering the hidden correlation between different features distinguish ML from the other possible methodologies to solve this problem. In [35], Bessa et al. were able to use regression models to predict the deformation parameters of some of the physical equations proposed by Chen et al. [31] and model the breast shape according to the degree of deformation defined by the user. Despite the promising results, it was limited by the usage of a completely synthetic dataset and by the dependence on previous knowledge of the physical equations of deformations. Here, the aim is to expand the described work by using real data, avoiding the usage of specific physical equations to model the breast deformations. Besides, to accomplish the requirement of providing a dataset with the demanded properties, an in-house dataset is generated using data from Magnetic Resonance Imaging (MRI) of real patients and an available breast cancer surgery simulator [24].

The rest of this paper is organized in five sections. Section 2 is focused on the design and construction of a dataset, with a brief description of the breast anatomy and a detailed explanation of the surgery simulator used to generate the dataset instances. In Section 3, the methodology followed in this work is described, from the feature engineering step to the machine learning approaches used. Implementation of the aforementioned regressions, together with numerical evaluations are explained in Section 4, indicating a promising prediction of the breast shape. Then, the complementary discussions will be expressed in Section 5. Finally, the explanations of the proposed methodology and generated dataset are wrapped up in Section 6, as conclusion.

## 2. Dataset for Learning Breast Healing Deformations

Historically, several factors have been identified to have a significant impact on the shape deformations caused by BCS, which can be grouped into patient, tumor and surgery related factors [4]. Hence, any model designed to predict breast deformations should take into account the influence of those characteristics. Since the main goal of this study is to learn the influence of different combinations of those factors, a large number of 3D data of patient’s breast before and after surgery (≈1 year) is mandatory. However, there is no available dataset that satisfies such requirements. As consequence, an in-house dataset was constructed, taking advantage of available MRI data, acquired before surgery and having some anatomical structures of interest annotated. Due to the lack of post-surgery data, the wound healing process was simulated using the BCS simulator proposed by Vavourakis et al. [24], which made the source code available for the scientific community.

In this Section, some insights on the breast anatomy and characteristics that condition shape deformations are provided, the framework designed by Vavourakis et al. [24] used to simulate BCS results is explained and the construction of the dataset used to develop the proposed methodology is detailed.

### 2.1. Breast Anatomy

**Figure 1:**
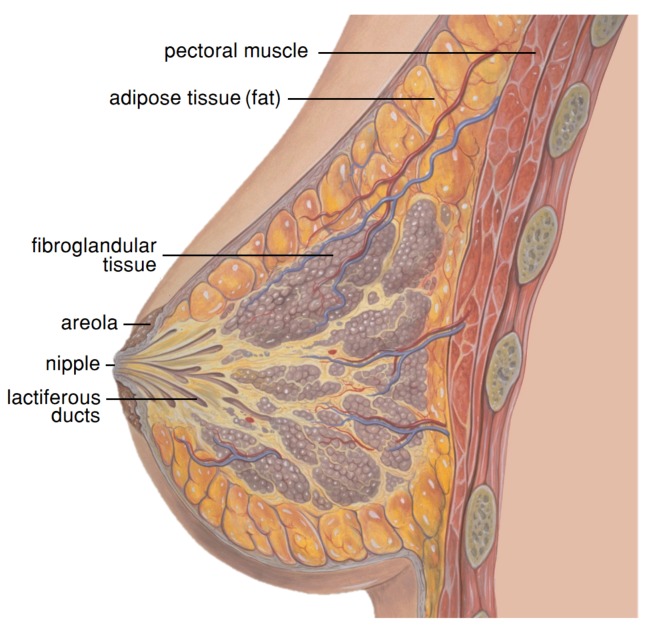
Breast anatomy: fibroglandular and fat tissues, which have distinct mechanical properties, compose most part of the breast - adapted from (https://commons.wikimedia.org/).

Breasts are important organs in a women’s body, whose primary role is related to sexual attraction and production of milk to nourish children. These highly deformable organs are located on the anterior and lateral parts of the chest, overlying the *pectoralis major* and *minor* muscles. With an heterogeneous structure (Figure 1), consisting of mammary glands (fibroglandular tissue) and adipose tissues (fat), breasts are firmly attached to the skin and underlying structures by fibrous bands referred to as Cooper’s ligaments. These suspensory ligaments provide the function of support, hold the breasts in place and contribute to determine the shape and contour of the breast [36]. Adipose tissue is fat-storing loose connective tissue, which determines the size of the breast, and mammary glands are modified sweat glands that are responsible for milk production.

The relative distribution of fibroglandular and fat tissues varies significantly depending on patient’s age, menstrual cycle, pregnancy/lactancy, hormone therapy and menopause, which affects the structure and morphology of the breast [36,37]. The ratio of these two types of tissues defines the breast density, which among other factors, can be related to the risk of developing breast cancer. In fact, women with a higher breast density are more likely to develop breast cancer [38]. Because fibroglandular and fat tissues have different mechanical behaviours, breast density also influences the aesthetical outcome of BCS, alongside with other breast characteristics, such as size and volume. Other factors that influence the deformations caused by BCS are the tumor size and location. Additionally, surgery-related factors such as the used technique, the placement of incisions, the volume of excised tissue or varying surgeon expertise lead to different types and extents of shape deformations [4]. Although the extent of the scar, color alterations or roughness of the treated breast also weight in its visual appearance, in this work only shape and geometry related deformations are modelled.

### 2.2. Wound Healing Simulator

**Figure 2:**
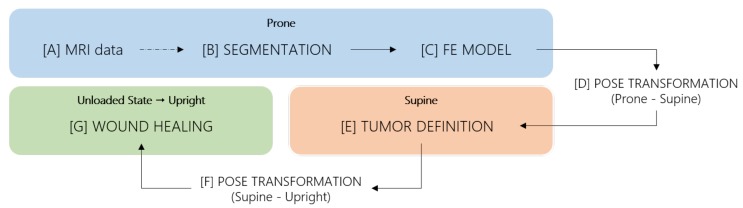
BCS simulation followed pipeline, as proposed by Vavourakis et al. [24].

Vavourakis et al. [24] proposed a BCS simulator that models the 3D post-surgical shape of the breast by coupling a physiological model of tissue recovery with a biomechanical model of pose estimation. The former predicts breast contraction caused by wound healing while the latter uses a Mooney-Rivlin biomechanical model of the breast to simulate deformations for different patient’s positions. The main stages of the surgery simulation (Figure 2) comprise the construction of a patient-specific Finite Element Model (FEM) of the breast, the definition of the tumor and the wound healing simulation itself, which uses the multiscale biomechanical model of wound healing described above.

**Figure 3:**
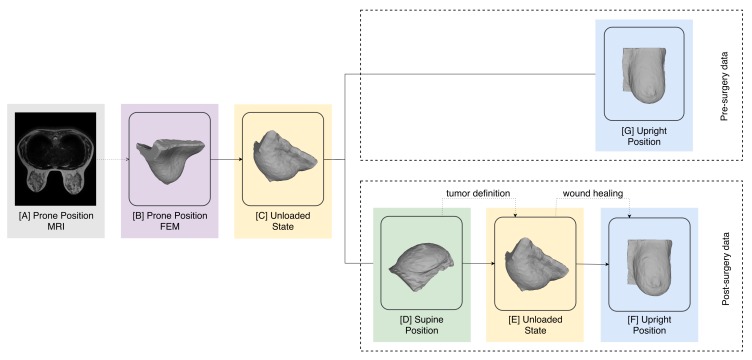
Pose transformation followed pipeline, as proposed by Vavourakis et al. [24].

Each of the wound healing pipeline stages are carried out with the patient in different positions and thus, some pose transformations are also accounted for (Figure 3). In fact, MRI is acquired with the torso facing down (prone position), surgery is performed with the patient facing up (supine position), the simulation occurs in an unloaded stage and surgery results are evaluated in the upright position.

#### 2.2.1. Finite Element Model

According to Vavourakis et al. [24], a 3D patient-specific biomechanical FEM of the breast can be created from MRI data (Figure 2A), that is segmented to delineate breast and background (Figure 2B). Fat and fibroglandular tissues can also be differentiated to create a more detailed FEM, in which distinct mechanical properties are assigned to elements according to the breast tissues they represent. Upon the segmentation of the structures of interest, a 2D surface mesh of FE that represents skin and a 3D mesh representing the interior of the breast shall be generated (Figure 2C).

#### 2.2.2. Pose Transformation

The FEM generated from MRI data represents the breast in the prone position because it assumes the configuration of the patient during MRI acquisition. Yet, during the surgery simulation work-flow, some stages require the model to represent the patient in different positions. As consequence, two explicit pose transformations occur (Figure 2D,F), and a third one takes place during the wound healing step (Figure 2G). In detail, MRI data is acquired with the patient lying in prone position (Figure 3A), but the upright position (Figure 3G) is the most suitable pose to evaluate the natural shape of breast and, consequently, its deformations. Thus, wound healing results are outputted in the upright position (Figure 3F). On the other hand, BCS is performed with the patient lying in supine position (Figure 3D), implying that any surgical planning activity, such as the definition of tumor characteristics and the volume to excise, should be done in this position. The multiscale model used for surgery simulation can estimate pose transformations by computing the unloaded state (a gravity free reference state) and re-applying gravity stress in the desired direction. As result, the FEM, constructed in prone position, is converted to an unloaded state (Figure 3C), from which both supine (Figure 3D) and pre-surgery upright position (Figure 3G) can be estimated. The tumor is defined in supine position (Figure 3D) and the FEM is again converted from supine to the unloaded state (Figure 3E), where the wound healing simulation is carried out. The simulation result (post-surgery data) is returned in upright position (Figure 3F). With this pose transformation pipeline, both pre- and post-surgery data are represented in the upright position, and can be directly compared for predicting shape deformations caused by BCS.

#### 2.2.3. Tumor Definition

The multiscale biomechanical model proposed by Vavourakis et al. [24] simulates the wound healing process taking as input the volume excised during surgery. This volume depends on the tumor position and size, and therefore, a virtual surgery has to occur in which all FE inside the volume to excise are re-labelled as damaged and assigned with different physiological and mechanical parameters. In [24], this virtual surgery is simulated in the supine position: the surgeon identifies the tumor position, defines the incision lines and outlines the incision path inside the breast. The excision volume is then approximated by a cylinder that contains the lesion and whose axis is perpendicular to the chest-wall, extending from the skin to the pectoral muscle. All FE contained inside this cylinder are assigned with damaged tissue properties.

### 2.3. Dataset Construction

To create the dataset, a subset of MRI data from the PICTURE project (http://www.vph-picture.eu/) was used. In detail, T1-weighted MRI image sets were used, containing approximately 60 axial slices each, with an average voxel resolution of 0.59×0.59×3 mm, (*x*, *y* and *z* axes, respectively). Taking advantage of the manually annotated structures, 3D point clouds (PCLs) of patient’s torsos were created using the breast contour, *Latissimus Dorsi* muscles and the pectoral muscle, as frontal, lateral and posterior boundaries, respectively. However, considering that the computational cost of the wound healing simulation is decreased if performed for each breast individually, instead of using the entire torso as done by Vavourakis et al. [24], the torso point cloud (PCL) was vertically divided with a plane defined along the sternum. Performing this division, individual breasts PCLs were obtained, which provide breast shape variability in the dataset. Any breast PCL holding visible MRI coil compression are discarded from the simulation because the deformation is not reversible with the multiscale biomechanical model.

After segmentation, each resulting breast PCL (≈1900 surface points) was converted to a 3D triangulated surface mesh to model the skin, using the Ball-Pivoting algorithm [39], in MeshLab [40]. The breast volume was next meshed in Gmsh [41], by inserting uniformly distributed points inside the object (≈2500 volume points), subsequently connecting them with tetrahedrons elements. To complete the creation of the FEM, distinct boundary conditions and material properties were assigned to the surface mesh - frontal surface, pectoral muscle (back surface), lateral limits, and top and bottom boundaries were defined - following the strategy proposed by Vavourakis et al. [24].

For the purpose of data augmentation, the number of dataset instances was increased by varying some input parameters related to breast and tumor characteristics, namely the breast density, and the tumor size and position, known to influence the aesthetical result after BCS.

To represent all categories of breast densities in the dataset, it was necessary to model other ratios of tissues than the ones represented in original MRI data, by varying the number of FE that are assigned with mechanical properties of fibroglandular and fat tissues. To avoid a segmentation step, the approach proposed by Del Palomar et al. [42] was used to simplify the structural complexity of the breast, by assigning a weighted average value of the mechanical properties of each tissue type to all elements of the model. Hence, the ratios defined by the American College of Radiologists (ACR) classification system - the Breast Imaging Reporting and Data System (BI-RADS^®^) [43] - can be considered for weighting the material property values described in [24], and represent several breast densities. This reporting system identifies 4 categories of breast density (A, B, C and D), which are described in Table 1. Following this strategy, the fibroglandular/fat ratios: A—10/90; B—35/65; C—60/40; D—85/15, were used to average material properties of each category, as detailed in Table 2.

**Figure 4:**
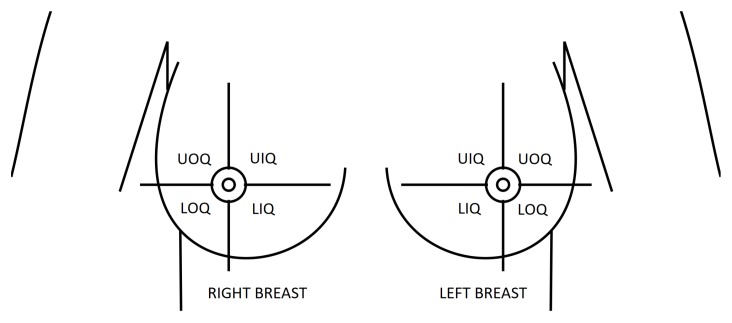
Breast quadrants definition: Upper-Outer (UOQ), Upper-Inner (UIQ), Lower-Outer (LOQ), Lower-Inner (LIQ) quadrants - adapted from (https://commons.wikimedia.org).

Besides the breast shape and composition, it is also important to characterize the tumor location and size. Since the relationship between tumor position and the aesthetical outcome is generally defined using breast quadrants to discretize tumor locations, tumors are randomly positioned inside each quadrant instead of choosing any position inside the breast, which assures the representativeness of the dataset by guarantying that there are instances of tumors in every quadrant. There are 4 quadrants defined by a vertical and horizontal division of the breast through the nipple and in the upright position (Figure 4): Upper-Outer or superolateral Quadrant (UOQ), Upper-Inner or superomedial Quadrant (UIQ), Lower-Outer or inferolateral Quadrant (LOQ), Lower-Inner or inferomedial Quadrant (LIQ) [44].

**Figure 5:**
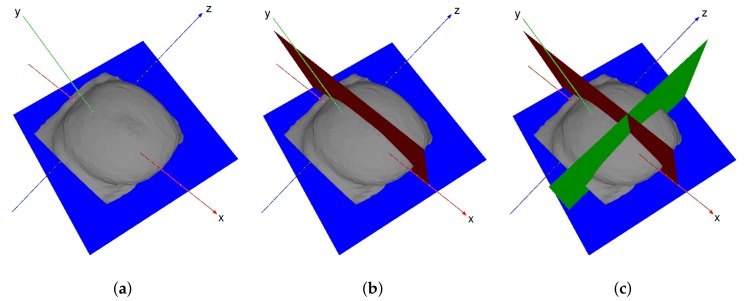
Definition of breast quadrants planes in supine position. (**a**) Pectoral plane; (**b**) Superior-Inferior plane (red); (**c**) Lateral-Medial plane (green).

The breast quadrants division is established in upright position. However, according to the surgery simulator pipeline (Figure 2), the tumor is defined in the supine position. Therefore, in an attempt to define quadrants in the horizontal position which correctly correspond the upright ones, the nipple position (manually annotated) is used as a reference point to compute the vertical and horizontal planes that define the quadrants boundaries. Three main planes are then sequentially defined, as seen in Figure 5. The first plane corresponds to the one along the pectoral muscle (Figure 5a) and is defined by its normal which is computed by the cross product of two vectors, defined by three corner points of the pectoral muscle (two on the top and one on the bottom). The second plane sets the superior-inferior boundary (red plane in Figure 5b) and is defined as parallel to the xy-plane (taking into account that the direction of the MRI acquisition is perpendicular to this plane), crossing the nipple. Finally, the third plane sets the lateral-medial boundary (green plane in Figure 5c), being perpendicular to the pectoral plane and crossing the nipple too.

**Figure 6:**
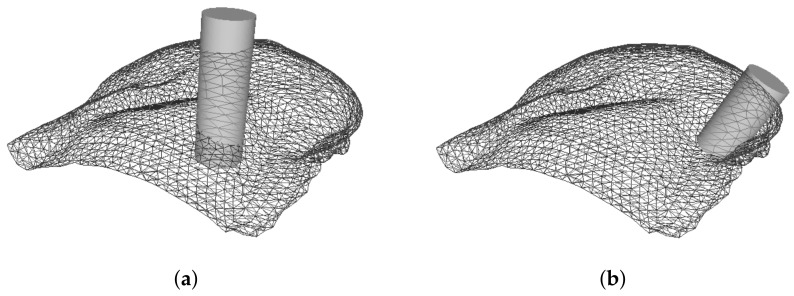
Alignment of a predefined cylinder (**a**) through the direction given by pectoral normal; in relation to the tumor location (**b**).

**Figure 7:**
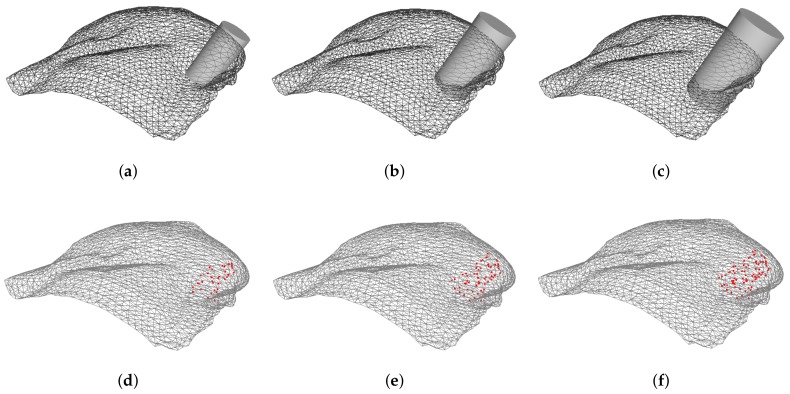
Definition of the cylinder to excise and the correspondent points labelled as damaged: small (**a**,**d**), medium (**b**,**e**) and large tumor sizes (**c**,**f**).

Having defined the quadrants, the tumor position is then randomly selected inside each quadrant - 3 spatial coordinates (*x*, *y* and *z*) are used to set the center of the tumor - and the excision volume is computed. Briefly, the line between the nearest point of the pectoral muscle and the tumor position sets the normal vector to the muscle, and a predefined cylinder (with a known radius, height and, consequently, volume) is aligned through this direction (Figure 6). Different tumor sizes (volumes) can then be modelled by varying the ratio between cylinder and breast volumes. Once the BCS protocol states that a breast tumor is eligible for BCS only if its removal do not require excision volumes higher than 20% of breast volume [45], cylinder volumes need to be limited to respect this threshold. In this study, excision volumes of 5%, 7.5% and 10% of the total breast volume were simulated, corresponding to three size categories: small, medium and large tumors, respectively (Figure 7a–c).

Once the cylinder is defined, the FE inside it are set as damaged (Figure 7d–f), and assigned the correspondent biomechanical and biochemical properties used by Vavourakis et al. [24].

**Figure 8:**
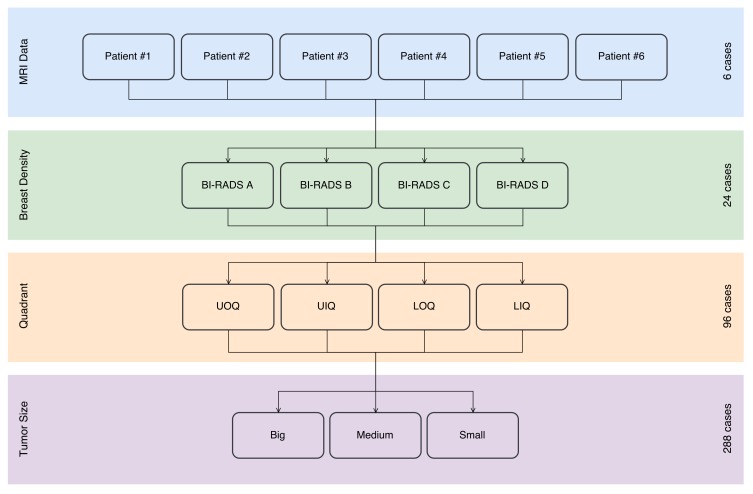
Sequential features combination used in dataset construction.

Figure 8 shows the main considerations made in the conception of the dataset. This dataset was built using 6 breast PCLs (obtained from MRI data), taking into account a uniform distribution of breast volume (2 small, 2 medium and 2 big breasts) and breast laterality (3 left and 3 right breasts), as described in Table 3. Dataset instances were created by sequentially defining 4 different breast densities for each breast selected before, according to BI-RADS^®^ reporting system (4×6=24 cases), then different quadrants for the tumor location (4×24=96 cases) and, finally, 3 different tumor sizes for each location (3×96=288 cases). In the end, the dataset sums up to a total of 288 cases representing all the possible combinations of the most prominent clinical factors reported to affect breast shape after BCS.

## 3. Methodology

The prediction of the post-surgery shape of the breast after surgical intervention is a complex task that requires modelling the influence of several factors on the aesthetic outcome of surgery. Approaches to model these deformations are typically based on biomechanical models. However, FEM takes longer than expectations, from hours on high-end machines, up to some days on normal computers used in clinics. In this work, an alternative strategy-based on machine learning techniques is proposed which overcomes the timing demands of biomechanical simulation, keeping most of the properties and characteristics of the breast.

### 3.1. Features

Feature extraction and representation is an important step in any machine learning task. Although clinical evidence suggests that a prediction model for breast deformation after BCS should take breast shape (laterality), volume and density into account, considering tumor characteristics such as quadrant (position) or size as inputs, such factors should be inspected in a more systematic way to confirm their influence and effects. Moreover, such analysis is important to suggest the best suited machine learning algorithms to model the problem at hand. For visual purposes, the feature investigation was constrained only to the breast surface.

#### 3.1.1. Breast Characteristics

**Figure 9:**
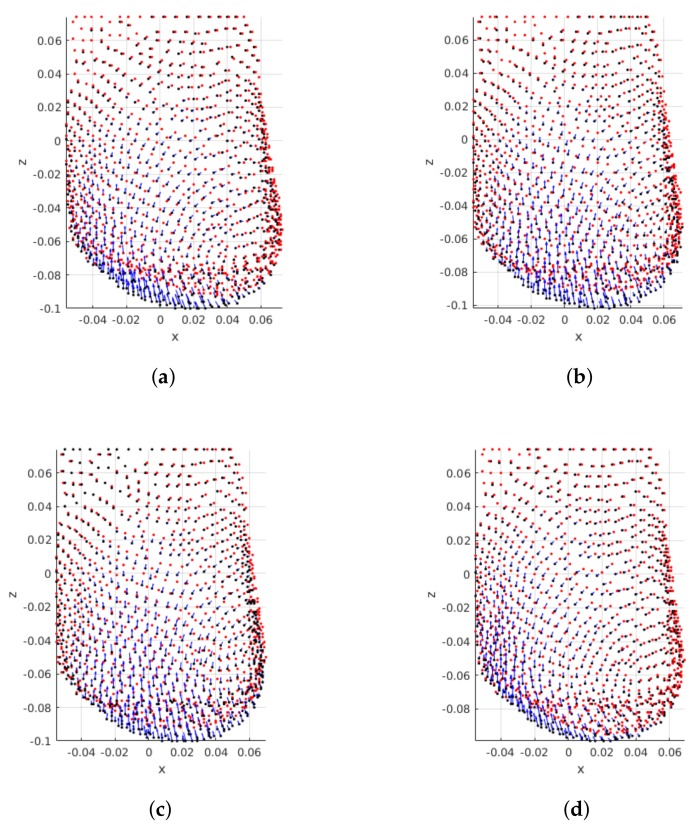
Influence of breast density on deformations: PCLs are shown for the same patient with variable breast density and fixed tumor position (UOQ) and size (small). Pre and post-surgical data on black and red, respectively. Blue arrows indicate displacement direction and magnitude. (**a**) BI-RADS A; (**b**) BI-RADS B; (**c**) BI-RADS C; (**d**) BI-RADS D.

Regarding breast characteristics, both density and shape are known to affect the extent of breast points displacements. Figure 9 shows the superposition of pre- and post-surgical breasts of the same patient, when different densities are modelled. The resulting plots show that breast density impacts the magnitude of points displacements: the magnitude of displacements decreases as the breast density increases. In fact, this was the expected behaviour, because denser breasts have a higher fraction of glandular tissue, which is less deformable than fat.

**Figure 10:**
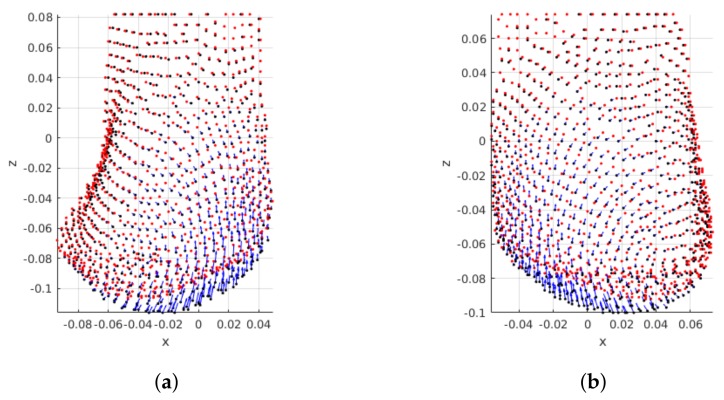
Influence of breast shape/laterality: PCLs are shown for two patients with BI-RADS A and the largest tumor positioned on UOQ. Pre- and post-surgical data on black and red, respectively. Blue arrows indicate displacement direction and magnitude. (**a**) Patient’s right breast; (**b**) Patient’s left breast.

As for breast laterality the mirroring of displacements can be seen when right and left breasts are compared (Figure 10). Although the magnitude of displacements is similar, the direction changes according to the breast laterality.

#### 3.1.2. Tumor Characteristics

**Figure 11:**
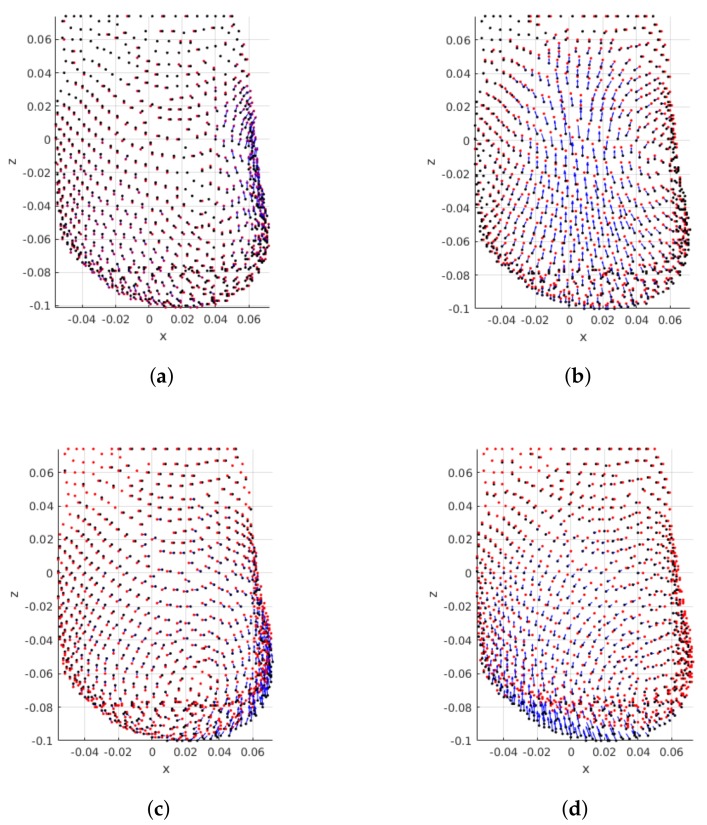
Influence of tumor position on deformations: PCLs are shown for the same patient with the tumor positioned in different breast quadrants/regions. Largest tumors are shown for BI-RADS A. Pre- and post-surgical data on black and red, respectively. Blue arrows indicate displacement direction and magnitude. (**a**) UIQ; (**b**) UOQ; (**c**) LIQ; (**d**) LOQ.

Figure 11 shows the effect of the tumor position on the displacements between pre- and post-surgical data. An interesting effect can be noticed: the distribution of displacements on the breast is dependent on the breast quadrant where the tumor is positioned. Larger displacements are centered around the tumor, vanishing as the distance to the tumor center increases. Hence, a feature space transformation might be helpful to describe the displacements distribution as function of the tumor position. In alternative, Euclidean and polar distance to tumor could benefit the modelling of the points displacements.

**Figure 12:**
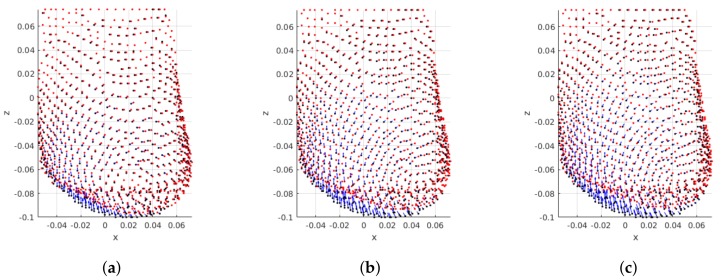
Influence of tumor size: PCLs are shown for the same patient with BI-RADS A, and tumor on region UOQ. Pre- and post-surgical data on black and red, respectively. Blue arrows indicate displacement direction and magnitude. (**a**) Small; (**b**) Medium; (**c**) Large.

Finally, the influence of the tumor size on the magnitude of breast displacement is shown in Figure 12. Results evidence that larger tumors cause larger breast displacements after surgery wound healing. In fact, this was the expected behaviour: after tumor removal the remaining breast tissues adapt to fill the left void. This results in breast contraction, which is a function of the excised volume.

#### 3.1.3. Feature Engineering

A brief look to Figure 9, Figure 10, Figure 11, Figure 12 denotes how different breast and tumor characteristics influence breast deformations after BCS and respective wound healing process. Breast density and tumor size have particular impact on the magnitude of the displacements, while the quadrant where the tumor is positioned and the breast laterality influences the distribution of those displacement. Therefore, one can expect that breast deformations can be modelled using the spatial coordinates of points, the distance of each point to the tumor position (the distance from a point perpendicular to the tumor cylinder), while accounting for categorical features such as breast density, tumor region, breast laterality and tumor size, as described in Table 4. Despite being appointed as an important clinical factor influencing the aesthetics of breasts after BCS, the volume of the breast is not explicitly listed. However, that information is implicitly covered due to the categorization of tumor sizes (expected excision volumes) which are defined as a percentage of the breast volume.

Definition of the features has been conducted aligned with the aforesaid expectations. The constructed feature list comprises three data attributes: points’ coordinates, points’ difference to the excised cylinder (both Euclidean and polar) and points’ distance to the excised cylinder as quantitative continuous, tumor size and breast density with categorical ordinal, and breast laterality and tumor region with categorical nominal attribute. The point coordinates simply expresses the location of each point in the 3D coordinate system, while the point coordinates difference feature reflects the difference between healthy points and the excised cylinder in each axis of the coordinate system. While the distance to the excised cylinder highlights the Euclidean distance from each healthy point, the polar distance to the excised cylinder expresses the same distance, but in polar notation. It is important to note that the coordinate difference and distance features for damaged points (inside the excised cylinder) are considered to be zero.

### 3.2. Regression Models

In this section, it will be explored solution taken into consideration the feature analysis above. The intention of exploiting machine learning in breast shape prediction is to estimate the point coordinates after surgery healing, taking as input the points positions before surgery, as well as breast and tumor characteristics. Since the points coordinates are continuous variables in 3D space, learning techniques providing regression methodologies are taken into consideration; however, prediction of the points coordinates poses a challenge to transfer breasts with different laterality or size, into the same coordinate system. Such circumstance can be prevented by re-formulating the demanded output from the regression. Instead of predicting the exact point coordinate, required displacement to translate a pre-surgery point to its post-surgery location can be predicted, alternatively. The post-surgery PCL is then attainable by applying predicted displacement on the pre-surgery PCL. Described in mathematical notation, the regression model can be expressed as in Equation (1):

(1) $$\left[\begin{array}{cc} P^{\;pre} & F\\ \vdots & \vdots \end{array}\right]\overset{f}{\to }\left[\begin{array}{c} disp^{\;pre\to post}\\ \vdots \end{array}\right],$$

where $P^{\;pre}$ is pre-surgery PCL, *F* is the feature list per instances (pre-surgery points), *f* is the demanded regression model, and finally $disp^{\;pre\to post}$ expresses the required displacements to convert pre-surgery PCL to post-surgery. Having predicted the displacement, predicted breast shape ($P^{pred}$) is attainable via Equation (2):

(2) $$P^{pre}+disp^{\;pre\to post}=P^{pred}$$

Looking back to the objective of predicting breast shape after BCS, the expected prediction should be performed considering the points of both pre- and post-surgery models, together with clinical features. Therefore, the possible learning approach to be proposed must be able to deal with large number of inputs (points), and correlate them with the features (clinical features). In ensemble learning the key idea is that different algorithms explore different search spaces and hypotheses, so composite systems could outperform single ones [46]. The strategy of exploiting ensemble learning methodology assures to gain improvement of not only the robustness, but also the performance of the learners via combining the votes of stronger single regressors combined to build the prediction model. As an ensemble learning approach, tree-based learners provide an appropriate framework (both in time and performance evaluations) to take part in training with large number of inputs. Therefore, within this research, tree-based learning methodologies are taken into consideration to perform the required regression in finding the predicted coordinate of breast shape.

Regression methods also can be categorized based on the number of their outputs. While single output regression are the most used ones, the internal correlation between their voters can be set such that they can generate multiple outputs. Therefore, taking the problem of predicting breast shape into account, regressors can be categorized in the two types of Multiple Input Single Output (MISO), or Multiple Input Multiple Output (MIMO). Both types of the aforesaid regressors are studied and evaluated in this work.

#### 3.2.1. Random Forests

Depending on the goal whether to decrease bias error and overfitting, or to decrease both bias and variance, bagging and boosting are generally used, respectively. Concentrating in the bagging, unstable models, i.e., models whose performance is sensible to small perturbations of the training set, are trained with different replicas of the training set, obtained with replacement to keep the same number of examples (bootstrap aggregation). Then, new examples are predicted by uniform voting between the regressors trained with different dataset replicas. A special variant of bagging applied to decision tress results in the RF method, which operates by constructing multiple decision trees with bagging and random selection of features at each split of each tree. This mechanism assures that the constructed trees become correlated with one or more features which are strong predictors [47].

In this study, the use of bagging is exploit by learning RF models. This method requires the optimization of the number of trees to use as forest, as well as the number of features considered during the construction of trees.

#### 3.2.2. Gradient Boosting Regression

The algorithm for Gradient Boosting Regression is a recasted adaptation of AdaBoost that employs boosting methods in regression trees [48]. The general idea is to compute a sequence of simple trees, where each successive tree is constructed for the prediction residuals of the preceding tree [48]. Minimization of the loss (residuals) of the model (or regressor) is pursued by adding weak learners using a gradient descent procedure. Therefore, three elements are considered directly in developing a regressor with gradient boosting: a loss function, a weak learner, and finally an additive model to add weak learners to minimize the loss function. Considering decision trees as the weak learner, they are added one at the time, while the existing trees are kept unchanged. To ensure the simplicity of the learned trees, it is common to assign specific constraints to control the growth of trees, for instance, defining a maximum limit for depth, or the number of leaf nodes.

The capability of optimization is granted to the trees since they are constructed with parameters to be modified in direction of reducing the total loss function of the regressor. A tree which reduces the total loss is added to the existing sequence of trees [49,50].

As a greedy algorithm, gradient boosting has overfitting potential in training data, quickly. Therefore, common techniques such as regularization, assists the performance of prediction by penalizing various parts of the algorithm. The discussed constraints on tree construction is a example of the regularization methods to control the greediness of gradient boosting [51].

#### 3.2.3. Multi-Output Regression

Regression models are normally characterized by only one output; however, taken into consideration the problem here presented, it make sense to think in a strategy based in MIMO regressors. In order to predict more than one output, it can be simple considered a regressor for each output, by concatenating several MISO regressors [52]; however, ignoring possible relationship among the constructed models could result in a drawback for the solution. A smarter solution is also suggested to construct several regressors not only by the input training data, but also by the possible internal relationships between them. In particular, this solution takes the advantage of constructing a MIMO regressor which is smaller than the size of those MISO regressors. It should be noted that the discussed solution leads to better predictions when there is a strong correlation between the features and the targets.

### 3.3. Summary

Through a comprehensive study between the pre- and post-surgery PCLs, the influence of each breast and tumor characteristics features were determined. Aligned with the objective of the current research to predict the shape of the breast after surgery, regression was taken into consideration as the main solution. Further discussions unveiled that the tree-based methodologies are capable enough to satisfy the input/output demands of the solution.

## 4. Results

The definition of training and testing sets is carried out with a careful approach, in which a patient leave-one-patient-out (LOPO) strategy is advised to obtain test and train subsets. In each split, all example data generated from the same input patient would be used for testing, and the remaining patient’s data would compose the training subset. In the particular case of having 6 patients, the LOPO strategy is followed to prevent overfitting, due to the similarities between the PCLs of a single patient. To assess the model performance through LOPO strategy and tuning parameters at the same time, a cross-validation approach was used in order to find the parameters’ which presents the best configuration of the model.

As there is a deterministic correspondence between the points of the predicted (as the source) and the post-surgery PCL (as the target) for each patient, the evaluation metric can be defined by the Euclidean *point-wise distance (p2p)*. Denoted in Equation (3), the point-wise distance evaluates the performance of the regression model, since it measures the amount of displacement of each pair of points regardless of the total PCL displacements. Less distance means the regression model predicted the coordinate of each point closer to its expected location.

(3) $$D^{p2p}=\frac{1}{N}\sum_{i=1}^{N}d\left(P_{i}^{\;source},P_{i}^{\;target}\right),$$

where *N* and *d* denote the number of points and Euclidean distance, respectively, and $P_{i}^{source}$ is the corresponding point of $P_{i}^{target}$. Not only point-wise, but also *global distance* can be calculated, as well. Unlike the point-wise, the global distance appraises the displacement between the two comparing sets in whole. That means the discussed metric gives an overview of the similarities between the source set with the target. The global measurement of the distance results in reporting two distances: from the source to target PCL and from the target PCL to the source. Closer reported distances signify the more similarity between the two PCLs.

(4) $$\left\{\begin{array}{c} D_{\;source\to target}^{\;global}=\frac{1}{N}\sum_{i=1}^{N}\min_{j}d\left(P_{i}^{\;source},P_{j}^{\;target}\right)\\ D_{\;target\to source}^{\;global}=\frac{1}{N}\sum_{i=1}^{N}\min_{j}d\left(P_{i}^{\;target},P_{j}^{\;source}\right) \end{array}\right.,$$

where the $P_{j}$ is the nearest point to $P_{i}$.

Since the deformation of breast is correlated with both internal and surface tissue (described in Section 2.2.3), both so-called points are considered during the training stage; however, numerical evaluation only comprises the analysis of the surface points.

Note that for the both presented metrics, the mean distance (μ), the standard deviation from the mean (σ) and the maximum distance (Max) between the comparing sets are reported as well. The maximum distance expresses the furthest distance between two corresponding points of the predicted and pre-surgery PCLs. Also, pre and post symbols stand for pre- and post-surgery PCLs, and pred denotes the predicted breast PCL (wherever it is needed the predictions are also compared visually with the post-surgery models).

Finally, the machine learning implementations for RF and GBT were accomplished in python 3, by using *scikit-learn* package [53] on a machine powered by intel${}^{®}$ Core i7${}^{®}$ at 3.2 GHz with 128 GB of memory (for cross validation). As long as the *scikit-learn* package includes multi-output regressor which is based on concatenation of individual regressors, the implementation of MOR was carried out with a package in *R*, called *Multivariate Random Forest* [54].

### 4.1. Random Forests

Following the approach of individual regressor for each axis, three RF regressors were trained. The cross-validation inner loop was set to optimize the number of estimators (trees), the maximum number of features in each tree, and the leaf size are of those parameters through {5,10,…,500}, {2,3,…,23}, and {1,2,…,5}, respectively.

The criterion to select the tuned parameters was chosen considering two objective functions (OF): the average, and the Hausdorff (maximum). Focusing on the average, the best set of tuned parameters are selected such that it minimizes the average distance between the predicted and the post-surgery models. The other OF which is based on the Hausdorff distance, intends to decrease the maximum distance between the points in each set of comparison of predicted and post-surgery PCLs, although the average distance may increase.

#### 4.1.1. PCL Sampling

**Figure 13:**
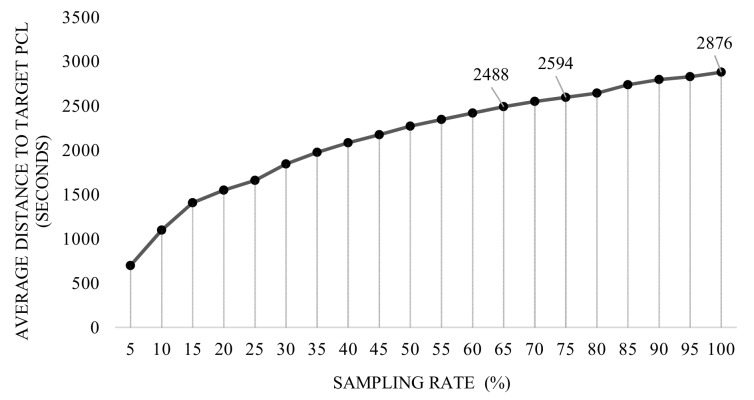
Impact of sampling breast PCL on the training time of RF.

**Figure 14:**
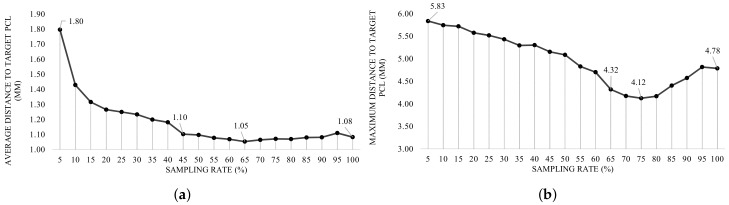
Impact of sampling breast PCL on the average (**a**) and Hausdorff (**b**) with respect to the pair-wise distance.

Theoretically in RF, inclusion of more points (more sampling rate) is thought to increment the gain of the regressor by declining the average distance between the predictions and the target, though, not only the training time continues its incremental trend, but also the aforesaid slope of the distance decelerates [55]. In this regard, a comprehensive study was designed to find an optimized sampling rate in the range of {5,10,…,100}, according to both distance error and training time. The timing complexity reported in Figure 13 depicts the aforesaid evolution as the training size set increases, as expected. Besides, shown in Figure 14a, considering the average OF, the declining slope of the distance error decreases in defiance of sampling rate, until it reaches to 65%. Although the difference of the reported distances between the rates of 45% and 65% is less than 0.50 mm, to satisfy the condition of the OF, it was decided to use the sampling rate correlated with the global minimum distance (65%).

With the same argument, and in accordance with the Hausdorff OF, the sampling rate of 75% was selected due to the least evaluated maximum distance, though the difference between the rates 75% and 65% is measured around 0.196 mm. Figure 14b depicts the evolution of maximum distance according to the Hausdorff OF. Considering the aforesaid sampling rates, numerical evaluations with respect to average and Hausdorff OFs are calculated and reported in Table 5 and Table 6, respectively. To evaluate the magnitude of the reported distances, an extra comparison is performed with the distance between the two comparing PCLs in case no method is applied (meaning that prediction data is exactly equal to the pre-surgery data). This comparison, so-called *baseline evaluation*, is reported in the last column of Table 5 and the last two columns of Table 6, for both the average and the Hausdorff OFs.

#### 4.1.2. Assigning Weights

Deep investigation of the trained RF reveals that the nature of problem demands to define different weights for the points belonging to healthy or damaged tissue. In this work a weight assigning strategy is followed in which the weights are initially assigned to the training instances and then they are updated iteratively in accordance to the distance from their correspondences in the target model. Thus, in each iteration, each point is assigned a weight that is proportional to the distance from its corresponding point of the target. This proposed iterative approach, called adaptive weighting, continues until either a fixed number of number of iteration is reached (in this case 100), or the OF is not satisfied within three consecutive iterations.

**Figure 15:**
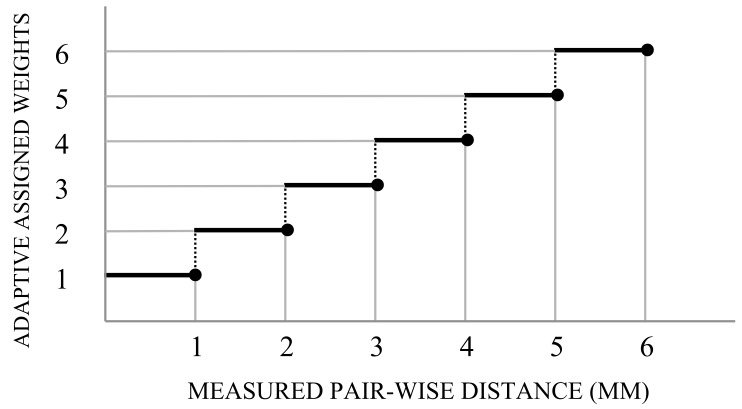
Assigning adaptive weight with respect to the range of the measured distances.

A glance to the results obtained in the previous section reveals that the distance for a point is near to 1 mm while the maximum distance is higher than 5 mm. Taken this in consideration we considered a weighting strategy by defining the values for the weights as the *ceiling* of the *point-wise distance (p2p)* in a range of 1 to 6, as shown in Figure 15.

Obtained results from evaluation show an interesting trend in decreasing both average and Hausdorff distances. Table 7 and Table 8 express the numerical evaluation based on average and Hausdorff OFs, respectively. Comparing with the best set of results (obtained by using RF with 65% sampling), a slight improvement is observed (1.048 mm for weighted RF vs. 1.052 mm for weightless). Note that both regressors are built using sampled dataset with the rate of 65%. Same decreasing trend is observed while the Hausdorff OF in considered (comparing 4.083 mm for weighted RF vs. 4.100 mm for weightless RF). This improvement certifies the assumption that the provision of a suitable weighting approach can lead the regression to predict post-surgery models with less distance evaluations. It should be noted that the weight assignment strategy has a significant impression to decrease the distance between predicted and post-surgery PCLs. Therefore, a new line of research is opened to investigate appropriate strategies to improve the prediction of breast shape using RF.

**Figure 16:**
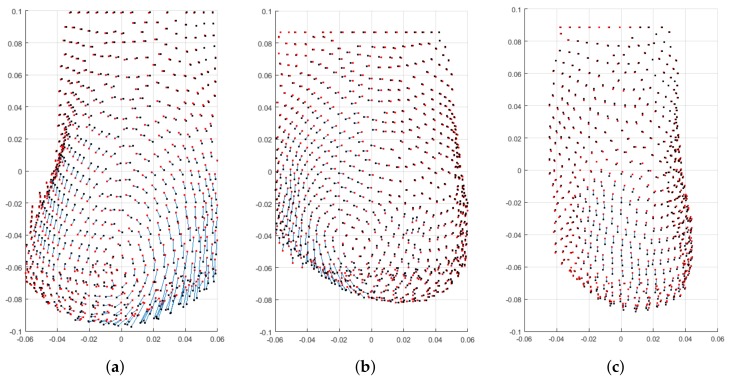
Visual evaluation: Three PCLs from dataset with different prediction distances. The post-surgery PCL is shown with red points while the predicted PCL is visualized with black points. The displacements between corresponding points are colored in blue. (**a**) Poor prediction, $D_{pred\to post}=1.624$ mm; (**b**) Fair prediction, $D_{pred\to post}=1.044$ mm; (**c**) Good prediction, $D_{pred\to post}=0.827$ mm.

Besides the reported numerical evaluation, visual comparisons of three predicted breasts are depicted in Figure 16. The depicted predictions have been evaluated with average pair-wise distance of 1.62 mm (Figure 16a) as a poor prediction, 1.044 mm (Figure 16b) as a fair prediction, and 0.827 mm (Figure 16c) as a good prediction.

#### 4.1.3. Feature Importance

Although the displacement of the points plays an important role in finding the regression, the used clinical features contribute in adjusting the amount of prediction required for each point of healthy or damaged region. The construction of trees in RF allows to report the importance of each feature. In this regard, clinical studies can be accompanied with the resulted features’ importance to highlight the ones which contribute more in the prediction. Based on the conducted analysis, the clinical features applied in the training procedure are studied to determine their level of importance to construct the regressor. Table 9 denotes the importance of features in percentage, for the regressors trained in each of the three axes. Additionally, for the features belonging to the same group, not only the individual importance, but also the grouped importance (average of individuals) is reported, as well.

From the obtained results it is observed that the point coordinates has the most significant effect in prediction and as expected, each coordinate point, individually, contribute more in their own axis. The second remarkable feature is the distance to tumor which reflects the Euclidean distance of each point to the tumor (represented as a cylinder). Such trait was also relevant to the features’ behavior observed in clinical analysis, since points in larger distances from the tumor region are deformed less (see Section 3.1.2). Another interesting observed trend is the impression of the *Z* axis on the importance of the features. While the breast is more affected in the *Z* axis with respect to the tumor location (see Section 3.1), it is expected that features related with it, are more represented on that axis. Reported analysis of the two features, *coordinate difference to the excised cylinder* and *polar distance to the excised cylinder* satisfy the expected hypothesis. Far from expectations, it can be deduced that the tumor size feature not only influences the breast final deformation less than other clinical features, but also shows an irrelevant behaviour compared with the tumor region or the breast density features.

Additionally, to complete the investigation of features on the evaluation results, each clinical feature is assessed individually with respect to its impression of the best obtained prediction. Table 10 reports the results of individual evaluations on the three studied clinical features including breast density, tumor size, and tumor region. By looking to the breast density, it is observed that the average distance error decreases with a direct relation to the amount of the adipose tissue (Table 10), as was previously observed in Section 3.1.1. It can be deduced, breasts with more fibro-glandular tissue, are less affected. The results reported in Table 9 signifies the weakest role of the tumor size among clinical features; however, the outcome presented in Table 10 provide more evidence to evaluate the contribution of this feature. It is observed that large tumors imposes more deformation, which is also an expected result, based on the results obtained in Section 3.1.2. Regarding tumour region, it is difficult to conclude anything relevant, since, by looking to the obtained results, the magnitude of the deformation seem independent on the quadrant.

### 4.2. Gradient Boosting Regression

As in RF, in GBR a cross validation is performed with LOPO strategy, the following range for each parameter of the model:number of estimators (trees), in the rage of {5,10,…,500}; the maximum number of features in each tree in {2,3,…,23}; the leaf size in {1,2,…,5}; and the learning rate in the range of {0.01,0.02,…,1}. Besides, the criterion which measures the quality of a split is set *friedman mse*. The numerical evaluation are reported in Table 11 and Table 12, for both the average and Hausdorff OFs.

Numerical comparisons indicate that the average *pair-wise distance* obtained by GBR was 1.326 mm and 1.285 for the average *global distance*. The performance of GBR should be investigated through the definition of the learners. To keep the learners weak, constraints were imposed on the number of leaves and the depth of the trees. The assigned constraints to maintain the trees small, have led to propagate error in the whole sequence of model, which is noticeable through the increase of the errors in comparison with RF; however, more research could be performed in future in order to improve the understanding of this behaviour.

By comparing with results obtained with RF, GBR presented worse results.

### 4.3. Multi-Output Regression

Contrarily to expectations, MOR presents poor results in both OF optimization, comparing to the RF. Numerical results of Table 13 and Table 14 express the performance of MOR with *pair-wise* and *global distances*.

Discussed in Section 4.1.3, some of the most important features, presents a high correlation of each individual axis with each specific direction. This fact might influence the MOR to present poor predictions. More experiments should be performed in the future, but by the obtained results we can say that the point coordinates could play an independent role in predicting the displacement of each axis.

### 4.4. Summary

In this section two machine learning methodologies were studied; RF and GBR. The influence of sampling the data was studied taken into account the performance of the algorithm in terms of computational time and error of the prediction. Additionally, the use of adaptive weights were shown to have positive influence in the prediction. In any case, it might be difficult to understand the magnitude of the error, due to the lack of comparative approach, which could lead to the weak understanding of the obtained results. For this reason, a Heuristic Model (HM) was designed, taken into account the feature analysis performed in Section 3.1. With this simple and heuristic method, we could understand how difficult is to achieve a good result without using complex models, taken into account only the knowledge of the problem.

The method comprises the computation of mean displacement (expressed in Equation 5) of each point in the PCL data. This is performed separately in each axis and in each quadrant independently.

(5) $${\bar{disp}}_{i\in \;\left\{x,y,z\right\}}^{\;q\in quadrants}=\frac{1}{n}\sum_{i=1}^{N}\left(P_{i}^{post}-P_{i}^{pre}\right)$$

where $P_{i}^{\;pre}$ and $P_{i}^{\;post}$ are corresponding points of pre- and post-surgery PCL, respectively, and *N* is the number of points in each PCL. The proposed HM follows the following strategy: the displacement of each point belonging to the healthy quadrants is computed based on the average displacement of each specific quadrant; for the points belonging to the unhealthy quadrant, the displacement is computed taken into account the average displacement of that quadrant, but multiplied both by {4,3,2,1} for the breast density ({A,B,C,D}, respectively), and by {3,2,1} for tumor size ({L,M,S}, respectively), following the knowledge obtained in Section 3.1 (see Equation (6)):

(6) $$P_{i}^{new}=\left\{\begin{array}{cc} P_{i}+{\bar{disp_{i}}}^{\;q} & P_{i}\in healthy\;breast\;quadrants\\ P_{i}+b\times s\times {\bar{disp_{i}}}^{\;q} & P_{i}\in quadrants\;which\;comprises\;the\;tumor \end{array}\right.,$$

where *b*, *s*, and ${\bar{disp_{i}}}^{\;q}$ are breast density, tumor size, and displacement of the equivalent breast quadrant, respectively, and finally, $p_{i}^{new}$ denotes the calculated post-surgery PCL. The presented results of Table 15 are obtained by this this approach.

By comparing this simple approach with the learning models presented previously, we can state that the magnitude of the error obtained with RF is acceptable. The framework designed in this work, composed by data, features and models, presented some simplification regarding the reference framework from Vavourakis et al. [24], which could lead to some errors on the prediction, but with not so high influence in the visual aspect. Some new directions for this line of research are already planned trying to improve the obtained results, which will be presented in Conclusions Section.

## 5. Discussion

Considering the characteristics of the problem, such as the type of features, and the demanded output, RF-based methodologies were primarily chosen as learning models for this problem. We started by studying the influence of data sampling for the performance of the model, and it was observed that less sampling generally leads to less errors on the prediction, but took to high computational time. Focusing on the *pair-wise distance*, the declining trend of errors stops when the sampling rate reaches to 65%. More sampling rates result in the errors oscillating in a the range of 0.1 mm. Although the difference errors between the distances in the range of 45% to 100% remains about 0.1 mm, the sampling rate with the minimum distance (1.048 mm) was chosen as the final sampling rate (65%). Same argument has been carried out to select the sampling rate of 75% when the OF is set to be Hausdorff distance. The minimum distance with respect to the Hausdorff OF is reported near to 4.022 mm.

The influence of weighting was also taken into account. Following the strategy of adaptive weight assignment, the points on training data PCLs were weighted based on the errors to the corresponding point in the target PCL. It should be noted that the trained model in each iteration is evaluated to determine the new weights for the next iteration. The obtained results highlight the effectiveness of using an adaptive weighting function, obtaining better results, even they are not so significant. Additional investigation should be performed in this part, in order to find the most suitable weighting function for this problem.

RF were compared with GBR methodology. As previously discussed, learners of the GBR was kept weak intentionally to refrain the greedy growth of the methodology. The imposed constraints kept the learner leaves less (or equal) than 3, and the learners depth less (or equal) than 5. Therefore, in both lines of OF, an increase of the distances were observed. However, it should be mentioned that the current strategy to consider the aforementioned constraints, limited the range of reported distances, in which the average and maximum distance in the two OFs approached to each other.

A MOR approach was also taken into consideration. Although the results showed that it fell behind the single output RF, this weak result might lay in the facts that not only the MOR trainer was unable to find a correlation between the points’ displacements of multiple coordinates, but also the features of different coordinates intruded the learner such that they cancel the impression of each others. The aforementioned hypothesis needs to be investigated more to determine the weak performance of MIMO methodology.

Finally, to close the discussion, it should be noted that all the regressors were design with a the same sampling rate. The RF with adaptive weights outperforms the other regressors with 1.048 mm and 4.083 mm for the average and the Hausdorff optimization, respectively. In the second rank, RF without consideration of adaptive weights stands with an average distance of 1.052 mm and maximum distance of 4.101 mm. Third and forth ranks were obtained by MOR (with average of 1.173 mm, and Hausdorff of 4.738 mm), and GBT(with average of 1.326 mm, and Hausdorff of 5.564 mm).

## 6. Conclusions and Future Work

In this study, the use of machine learning techniques to predict the breast deformation after BCS is developed to use real MRI data of patients without depending on the knowledge of physical equations to describe the deformations.

To overcome the nonexistence of dataset suited for learning breast healing deformations, an in-house dataset was generated using MRI data from real patients combined with a multiscale biomechanical and biochemical models to simulate post-surgical breast shape. Several clinical features that might impact breast healing deformations, including breast density, tumor region, and tumor size, were considered in the creation of the dataset that is representative of the population distributions.

The learning process was divided into two main tasks: feature inspection and model selection. In the first task, the complex interplay of clinical features for conditioning the breast shape after surgery healing was untangled by comparing the influence of different sets of features in the deformed breast after resulted from BCS. Here, it was concluded that breast density had the highest impact on the breast displacements while the quadrant where comprises the tumor was determining to predict the directions of shape adjustments. Further analyses of the importance of features resulted from learning process reinforced the experimental findings. In the model selection task, several regression methodologies, including Random Forest, Gradient Boosting Regression, and Multi-Output Regression were studied. Beside the learning model, a heuristic model was also proposed to validate the veracity of learning methodologies and understand the magnitude of the distances. Additional investigations were conducted with respect to sample PCL data, as well as a complementary study to assign different weights to the training instances, which resulted in predictions with slightly better evaluations. The numerical evaluations with the pair-wise and global distances indicated that the RF regressor constructed with the adaptive weighted training set outperformed MOR and GBR in both lines of average and Hausdorff OFs.

Improvement of results by assigning adaptive weights opens a new line of investigation for the future work, in order to define better mechanisms to improve the performance of regressor. Additionally, regression based on GBR can be improved by re-defining new learners and putting better constraints to keep the learners weak enough to avoid overfitting. Besides, more investigations are needed to study the correlations between the features in order to provide more evidence to the hypothesis of poor performance of MOR. As an alternative solution, it is worth studying a combined methodology of predicting breast deformation with machine learning techniques followed by some final steps of the biomechanical models; hence the required computational time for the biomechanical models is decreased since the predecessor machine learning methodology has provided a partial solution. Last but not least, new learning methodologies such as convolution neural network regression, and deep geometric learning can be investigated, as well as performing the prediction of breast shape directly on the 3D surface data.

## Figures and Tables

**Table 1:** BI-RADS^®^ breast density description [43].

Density Categories	% Fibroglandular Tissue	Description
A	<25%	Almost entirely fatty breast
B	25–50%	Scattered areas of fibroglandular density
C	50–75%	Heterogeneously dense breast
D	>75%	Extremely dense breast

**Table 2:** Combination of tissues biomechanical properties for each density category. $c_{1}$ and $c_{2}$ are parameters of the Mooney-Rivlin biomechanical model, and $\rho_{0}$ is the material density. The reference values from Vavourakis et al. [24] were used.

BI-RADS^®^	Biomechanical Properties		
	$c_{1}$ (Pa)	$c_{2}$ (Pa)	$\rho_{0}$ (kg·m${}^{-3}$)
A	0.1 × 120 + 0.9 × 80 = 84	0	0.1 × 1020 + 0.9 × 910 = 921
B	0.35 × 120 + 0.65 × 80 = 94	0	0.35 × 1020 + 0.65 × 910 = 948.5
C	0.6 × 120 + 0.4 × 80 = 104	0	0.6 × 1020 + 0.4 × 910 = 976
D	0.85 × 120 + 0.15 × 80 = 114	0	0.85 × 1020 + 0.15 × 910 = 1003.5

**Table 3:** Characterization of breast MRI data used for data augmentation: size, volume and laterality.

Breast Characteristics	Patient #1	Patient #2	Patient #3	Patient #4	Patient #5	Patient #6
Size	Small	Medium	Large	Medium	Large	Small
Volume (mm${}^{3}$)	495,948	802,661	1,314,990	1,052,500	1,202,500	559,677
Laterality	Left	Left	Right	Left	Right	Right

**Table 4:** Description of features used in the regression models to predict breast deformations after BCS.

Features	ID	Type	Space	Description			
Point’s coordinate	$p_{x}$	Quantitative Continuous	$\in {I\;R}^{3}$	Breast points coordinates in 3D space. The center of geometry of PCLs should be translated to the origin.
	$p_{y}$						
	$p_{z}$						
Coordinate difference to the excised cylinder	$disp_{x}$	Quantitative Continuous	$\in {I\;R}^{3}$	Difference of each healthy point (signed) of pre-surgery PCL to the excised cylinder
	$disp_{y}$						
	$disp_{z}$						
Distance to the excised cylinder	$d_{x,y,z}$	Quantitative Continuous	∈IR	Euclidean distance of each point of pre-surgery PCL to the excised cylinder			
Polar distance to the excised cylinder	ρ	Quantitative Continuous	$\in {I\;R}^{3}$	Polar difference of each healthy point (signed) of pre-surgery PCL to the excised cylinder
	ϕ						
	*z*						
Tumor Size	$s_{1}$	Categorical Ordinal	100	Defines the size of tumor (5%, 7.5%, or 10% of total breast volume)
	$s_{2}$		010			
	$s_{3}$		001				
Breast Laterality	*R*	Categorical Nominal	1	Indicates the laterality of breast (right or left)
	*L*		0				
Breast Density	*A*	Categorical Ordinal	1000	Determines breast density level (A, B, C, or D)
	*B*		0100			
	*C*		0010			
	*D*		0001				
Tumor Region	$R_{1}$	Categorical Nominal	1000	Specifies the region of breast with the tumor (UOQ,UIQ, LOQ, or LIQ)
	$R_{2}$		0100			
	$R_{3}$		0010			
	$R_{4}$		0001				

**Table 5:** Numerical evaluation: Pair-wise distance (in mm) based on the both average and Hausdorff OFs for training set sampled with 65%, and 75%, respectively. Also, the last column denotes the evaluation of dummy method.

	Average-Based OF	Hausdorff-Based OF	Baseline Evaluation
	$D^{p2p}$	$D^{p2p}$	$D^{p2p}$
μ	1.052	1.146	2.206
σ	0.920	0.934	1.920
Max	5.210	4.101	8.410

**Table 6:** Numerical evaluation: Global distance (in mm) based on both average and Hausdorff OFs for training set sampled with 65%, and 75%, respectively. The last two columns denote the evaluation of the dummy method.

	Average-Based OF		Hausdorff-Based OF		Baseline Evaluation	
	$D_{\mathrm{pred}\to \mathrm{post}}^{\;\mathrm{global}}$	$D_{\mathrm{post}\to \mathrm{pred}}^{\;\mathrm{global}}$	$D_{\mathrm{pred}\to \mathrm{post}}^{\;\mathrm{global}}$	$D_{\mathrm{post}\to \mathrm{pred}}^{\;\mathrm{global}}$	$D_{\mathrm{pre}\to \mathrm{post}}^{\;\mathrm{global}}$	$D_{\mathrm{post}\to \mathrm{pre}}^{\;\mathrm{global}}$
μ	0.973	0.970	1.091	1.071	1.758	1.731
σ	0.787	0.748	0.825	0.761	1.333	1.277
Max	4.926	4.814	4.025	4.010	6.512	6.317

**Table 7:** Numerical evaluation: Pair-wise distance (in mm) between predicted PCLs and post-surgery models using RF with adaptive weights. The training set is sampled with rate of 65% and 75% due to average and Hausdorff OFs, respectively.

	Average-Based OF	Hausdorff-Based OF	Baseline Evaluation
	$D^{p2p}$	$D^{p2p}$	$D^{p2p}$
μ	1.048	1.189	2.206
σ	0.905	0.981	1.920
Max	5.240	4.083	8.410

**Table 8:** Numerical evaluation: Global distance (in mm) between predicted PCLs and post-surgery models using RF with adaptive weights. The training set is sampled with rate of 65% and 75% due to average and Hausdorff OFs, respectively.

	Average-Based OF		Hausdorff-Based OF		Baseline Evaluation	
	$D_{\mathrm{pred}\to \mathrm{post}}^{\;\mathrm{global}}$	$D_{\mathrm{post}\to \mathrm{pred}}^{\;\mathrm{global}}$	$D_{\mathrm{pred}\to \mathrm{post}}^{\;\mathrm{global}}$	$D_{\mathrm{post}\to \mathrm{pred}}^{\;\mathrm{global}}$	$D_{\mathrm{pre}\to \mathrm{post}}^{\;\mathrm{global}}$	$D_{\mathrm{post}\to \mathrm{pre}}^{\;\mathrm{global}}$
μ	0.961	0.951	1.124	1.094	1.758	1.731
σ	0.951	0.861	0.958	0.937	1.333	1.277
Max	5.182	5.178	4.022	3.980	6.512	6.317

**Table 9:** Features’ importance (both individual and grouped) measured in % for RF with adaptive weights.

Features	ID	Trained Model for *X*		Trained Model for *Y*		Trained Model for *Z*	
		Individual (%)	Group (%)	Individual (%)	Group (%)	Individual (%)	Group (%)
Points’ coordinate	$p_{x}$	8.39	7.03	6.94	7.67	3.75	6.25
	$p_{y}$	7.46		8.37		6.93	
	$p_{z}$	5.25		7.68		8.08	
Coordinate difference to the excised cylinder	$disp_{x}$	1.17	1.73	0.90	1.19	1.37	1.75
	$disp_{y}$	1.46		1.25		1.57	
	$disp_{z}$	2.57		1.39		2.32	
Distance to the excised cylinder	$D_{x,y,z}$	8.23	8.23	8.09	8.09	8.00	8.00
Polar distance the the excised cylinder	ρ	0.11	0.42	0.04	0.47	0.29	0.70
	ψ	0.48		0.66		0.82	
	*z*	0.68		0.70		1.00	
Tumor Size	$s_{1}$	2.79	2.99	1.93	3.31	2.67	3.19
	$s_{2}$	3.03		2.21		3.21	
	$s_{3}$	3.16		5.79		3.69	
Breast Laterality	*R*	4.21	3.81	3.72	3.75	4.12	4.16
	*L*	3.42		3.79		4.19	
Breast Density	$B_{1}$	7.13	6.08	6.29	6.74	5.44	6.23
	$B_{2}$	7.00		7.30		6.35	
	$B_{3}$	5.10		6.51		6.44	
	$B_{4}$	5.11		6.87		6.69	
Tumor Region	$R_{1}$	4.77	5.95	4.44	5.81	5.97	5.76
	$R_{2}$	6.77		4.53		5.51	
	$R_{3}$	6.80		5.06		5.72	
	$R_{4}$	4.94		5.50		5.85	

**Table 10:** Individual evaluation of the clinical features (breast density, tumor size, and tumor region) with respect to the adaptive weight RF in scope of the average OF.

	Breast Density				Tumor Size			Tumor Region			
	A	B	C	D	S	M	L	UOQ	UIQ	LOQ	LIQ
μ	1.052	1.050	1.048	1.044	1.045	1.049	1.051	1.052	1.049	1.051	1.042
σ	0.931	0.910	0.890	0.891	0.900	0.905	0.911	0.920	0.901	0.910	0.891
Max	5.257	5.256	5.218	5.230	5.178	5.233	5.310	5.291	5.206	5.273	5.191

**Table 11:** Numerical evaluation: Pair-wise distance (in mm) between predicted PCLs and post-surgery models using GBR. The training set is sampled with rate of 65% and 75% due to average and Hausdorff OFs, respectively.

	Average-Based OF	Hausdorff-Based OF	Baseline Evaluation
	$D^{p2p}$	$D^{p2p}$	$D^{p2p}$
μ	1.326	1.631	2.206
σ	0.943	1.026	1.920
Max	5.933	5.564	8.410

**Table 12:** Numerical evaluation: Global distances (in mm) between predicted PCLs and post-surgery models using GBR. The training set is sampled with rate of 65% and 75% due to average and Hausdorff OFs, respectively.

	Average-Based OF		Hausdorff-Based OF		Baseline Evaluation	
	$D_{\mathrm{pred}\to \mathrm{post}}^{\;\mathrm{global}}$	$D_{\mathrm{post}\to \mathrm{pred}}^{\;\mathrm{global}}$	$D_{\mathrm{pred}\to \mathrm{post}}^{\;\mathrm{global}}$	$D_{\mathrm{post}\to \mathrm{pred}}^{\;\mathrm{global}}$	$D_{\mathrm{pre}\to \mathrm{post}}^{\;\mathrm{global}}$	$D_{\mathrm{post}\to \mathrm{pre}}^{\;\mathrm{global}}$
μ	1.287	1.269	1.604	1.590	1.758	1.731
σ	0.928	0.906	0.985	0.972	1.333	1.277
Max	5.735	5.701	5.439	5.394	6.512	6.317

**Table 13:** Numerical evaluation: Pair-wise distance (in mm) between predicted PCLs and post-surgery models using MOR. The training set is sampled with rate of 65% and 75% due to average and Hausdorff OFs, respectively.

	Average-Based OF	Hausdorff-Based OF	Baseline Evaluation
	$D^{p2p}$	$D^{p2p}$	$D^{p2p}$
μ	1.173	1.330	2.206
σ	0.993	1.022	1.920
Max	5.746	4.738	8.410

**Table 14:** Numerical evaluation: Global distances (in mm) between predicted PCLs and post-surgery models using MOR. The training set is sampled with rate of 65% and 75% due to average and Hausdorff OFs, respectively.

	Average-Based OF		Hausdorff-Based OF		Baseline Evaluation	
	$D_{\mathrm{pred}\to \mathrm{post}}^{\;\mathrm{global}}$	$D_{\mathrm{post}\to \mathrm{pred}}^{\;\mathrm{global}}$	$D_{\mathrm{pred}\to \mathrm{post}}^{\;\mathrm{global}}$	$D_{\mathrm{post}\to \mathrm{pred}}^{\;\mathrm{global}}$	$D_{\mathrm{pre}\to \mathrm{post}}^{\;\mathrm{global}}$	$D_{\mathrm{post}\to \mathrm{pre}}^{\;\mathrm{global}}$
μ	1.130	1.114	1.301	1.286	1.758	1.731
σ	0.935	0.903	0.983	0.966	1.333	1.277
Max	5.695	5.623	4.682	4.621	6.512	6.317

**Table 15:** Numerical evaluation: Point-wise and global distance (in mm) between predicted and post-surgery PCLs using heuristic model. The training set is sampled with rate of 65%.

	Average OF		
	$D^{p2p}$	$D_{\mathrm{pred}\to \mathrm{post}}^{\;\mathrm{global}}$	$D_{\mathrm{post}\to \mathrm{pred}}^{\;\mathrm{global}}$
μ	1.634	1.522	1.503
σ	1.496	1.193	1.129
Max	5.763	5.326	5.136

## Abbreviations

The following abbreviations are used in this manuscript:

2D	2 Dimensional
3D	3 Dimensional
ACR	American College of Radiologists
BCS	Breast Conserving Surgery
BI-RADS	Breast Imaging Reporting and Data System
FE	Finite Elements
FEM	Finite Element Model
GBR	Gradient Boosting Regression
LIQ	Lower-Inner Quadrant
LOPO	Leave One Patient Out
LOQ	Lower-Outer or inferolateral Quadrant
MIMO	Multiple Input Multiple Output
MISO	Multiple Input Single Output
MOR	Multi-Output Regression
MRI	Magnetic Resonance Image
OF	Objective Function
PCL	Pointcloud
RF	Random Forests
UIQ	Upper-Inner Quadrant
UOQ	Upper-Outer Quadrant

## References

[1] American Cancer Society (2014). Breast Cancer Detailed Guide.

[2] Gomes N.S., Silva S.R.d. (2013). Avaliação da autoestima de mulheres submetidas à cirurgia oncológica mamária. Text Context Nurs..

[3] Sakorafas G.H. (2001). Breast cancer surgery—Historical evolution, current status and future perspectives. Acta Oncol..

[4] Cardoso M.J., Oliveira H.P., Cardoso J.S. (2014). Assessing cosmetic results after breast conserving surgery. J. Surg. Oncol..

[5] Hill-Kayser C.E., Vachani C., Hampshire M.K., Di Lullo G.A., Metz J.M. (2012). Cosmetic outcomes and complications reported by patients having undergone breast-conserving treatment. Int. J. Radiat. Oncol. Biol. Phys..

[6] Aerts L., Christiaens M., Enzlin P., Neven P., Amant F. (2014). Sexual functioning in women after mastectomy versus breast conserving therapy for early-stage breast cancer: A prospective controlled study. Breast.

[7] Kim M.K., Kim T., Moon H.G., Jin U.S., Kim K., Kim J., Lee J.W., Kim J., Lee E., Yoo T.K. (2015). Effect of cosmetic outcome on quality of life after breast cancer surgery. Eur. J. Surg. Oncol..

[8] Tőkés T., Torgyík L., Szentmártoni G., Somlai K., Tóth A., Kulka J., Dank M. (2015). Primary systemic therapy for breast cancer: Does the patient’s involvement in decision-making create a new future?. Patient Educ. Couns..

[9] Oliveira H.P., Cardoso J.S., Magalhães A., Cardoso M.J. (2013). Methods for the Aesthetic Evaluation of Breast Cancer conservation treatment: A technological review. Curr. Med. Imaging Rev..

[10] Garbey M., Thanoon D., Bass B. (2011). Multi-Scale modeling in computational surgery: Application to Breast conservative therapy. J. Serbian Soc. Comput. Mech..

[11] De Heras Ciechomski P., Constantinescu M., Garcia J., Olariu R., Dindoyal I., Le Huu S., Reyes M. (2012). Development and implementation of a web-enabled 3D consultation tool for Breast augmentation surgery based on 3D-Image reconstruction of 2D pictures. J. Med. Int. Res..

[12] Eiben B., Han L., Hipwell J., Mertzanidou T., Kabus S., Buelow T., Lorenz C., Newstead G.M., Abe H., Keshtgar M. Biomechanically guided prone-to-supine image registration of breast MRI using an estimated reference state. Proceedings of the IEEE 10th International Symposium on Biomedical Imaging.

[13] Han L., Hipwell J.H., Eiben B., Barratt D., Modat M., Ourselin S., Hawkes D.J. (2014). A nonlinear biomechanical model based registration method for aligning prone and supine mr breast images. IEEE Trans. Med. Imaging.

[14] Rajagopal V. (2007). Modelling Breast Tissue Mechanics under Gravity Loading. Ph.D. Thesis.

[15] Hipwell J.H., Vavourakis V., Han L., Mertzanidou T., Eiben B., Hawkes D.J. (2016). A review of biomechanically informed breast image registration. Phys. Med. Biol..

[16] Mertzanidou T., Hipwell J., Johnsen S., Han L., Eiben B., Taylor Z., Ourselin S., Huisman H., Mann R., Bick U. (2014). MRI to X-ray mammography intensity-based registration with simultaneous optimisation of pose and biomechanical transformation parameters. Med. Image Anal..

[17] Hopp T., Dietzel M., Baltzer P.A., Kreisel P., Kaiser W.A., Gemmeke H., Ruiter N.V. (2013). Automatic multimodal 2D/3D breast image registration using biomechanical FEM models and intensity-based optimization. Med. Image Anal..

[18] Shih T.C., Chen J.H., Liu D., Nie K., Sun L., Lin M., Chang D., Nalcioglu O., Su M.Y. (2010). Computational simulation of breast compression based on segmented breast and fibroglandular tissues on magnetic resonance images. Phys. Med. Biol..

[19] Sturgeon G.M., Kiarashi N., Lo J.Y., Samei E., Segars W.P. (2016). Finite-element modeling of compression and gravity on a population of breast phantoms for multimodality imaging simulation. Med. Phys..

[20] Azar F.S., Metaxas D.N., Schnall M.D. (2001). A deformable finite element model of the breast for predicting mechanical deformations under external perturbations. Acad. Radiol..

[21] Carter T.J. (2009). Biomechanical Modelling of the Breast for Image-Guided Surgery. Ph.D. Thesis.

[22] Carter T.J., Tanner C., Crum W., Beechey-Newman N., Hawkes D. (2006). A framework for image-guided breast surgery. International Workshop on Medical Imaging and Virtual Reality.

[23] Garbey M., Bass B.L., Berceli S. (2012). Multiscale mechanobiology modeling for surgery assessment. Acta Mech. Sin..

[24] Vavourakis V., Eiben B., Hipwell J.H., Williams N.R., Keshtgar M., Hawkes D.J. (2016). Multiscale Mechano-Biological Finite Element Modelling of Oncoplastic Breast Surgery—Numerical Study towards Surgical Planning and Cosmetic Outcome Prediction. PLoS ONE.

[25] Rajagopal V., Nielsen P.M.F., Nash M.P. (2010). Modeling breast biomechanics for multi-modal image analysis-successes and challenges. Wiley Interdiscip. Rev. Syst. Biol. Med..

[26] Bardinet E., Cohen L.D., Ayache N. (1998). A Parametric Deformable Model to Fit Unstructured 3D Data. Comput. Vis. Image Underst..

[27] Rueckert D., Sonoda L.I., Hayes C., Hill D.L.G., Leach M.O., Hawkes D.J. (1999). Nonrigid Registration using free-form deformations: Application to breast MR images. IEEE Trans. Med. Imaging.

[28] Gallo G., Guarnera G.C., Catanuto G., Pane F. Parametric representation of human breast shapes. Proceedings of the IEEE International Workshop on Medical Measurements and Applications, MeMeA 2009.

[29] Lee A.W.C., Schnabel J.A., Rajagopal V., Nielsen P.M.F., Nash M.P. (2010). Breast image registration by combining finite elements and free-form deformations. Digital Mammography.

[30] Pernes D., Cardoso J.S., Oliveira H.P. Fitting of superquadrics for breast modelling by geometric distance minimization. Proceedings of the 2014 IEEE International Conference on Bioinformatics and Biomedicine.

[31] Chen D.T., Kakadiaris I.a., Miller M.J., Loftin R.B., Patrick C. (2000). Modeling for Plastic and Reconstructive Breast Surgery. Medical Image Computing and Computer-Assisted Intervention—MICCAI 2000.

[32] Seo H., Cordier F., Hong K. (2007). A breast modeler based on analysis of breast scan. Comput. Animat. Virtual Worlds.

[33] Gallo G., Guarnera G.C., Catanuto G. Human Breast Shape Analysis Using PCA. Proceedings of the Third International Conference on Bio-inspired Systems and Signal Processing.

[34] Kim Y., Lee K., Kim W. (2008). 3D virtual simulator for breast plastic surgery. Comput. Animat. Virtual Worlds.

[35] Bessa S., Zolfagharnasab H., Pereira E., Oliveira H.P. (2017). Prediction of Breast Deformities: A Step Forward for Planning Aesthetic Results After Breast Surgery. Pattern Recognition and Image Analysis.

[36] Ramião N.G., Martins P.S., Rynkevic R., Fernandes A.A., Barroso M., Santos D.C. (2016). Biomechanical properties of breast tissue, a state-of-the-art review. Biomech. Model. Mechanobiol..

[37] Thanoon D., Garbey M., Bass B.L., Garbey M., Bass B.L., Berceli S., Collet C., Cerveri P. (2014). Computational Modeling of Breast Conserving Surgery (BCS) Starting from MRI Imaging. Computational Surgery and Dual Training.

[38] Jennifer A., Harvey M.D., Viktor E.B. (2004). Quantitative Assessment of Mammographic Breast Density: Relationship with Breast Cancer Risk. Radiology.

[39] Bernardini F., Mittleman J., Rushmeier H., Silva C., Taubin G. (1999). The ball-pivoting algorithm for surface reconstruction. IEEE Trans. Vis. Comput. Graph..

[40] Cignoni P., Callieri M., Corsini M., Dellepiane M., Ganovelli F., Ranzuglia G., Scarano V., Chiara R.D., Erra U. (2008). MeshLab: An Open-Source Mesh Processing Tool.

[41] Geuzaine C., Remacle J.F. (2009). Gmsh : A 3-D finite element mesh generator with built-in pre- and post-processing facilities. Int. J. Numer. Methods Eng..

[42] Del Palomar A.P., Calvo B., Herrero J., López J., Doblaré M. (2008). A finite element model to accurately predict real deformations of the breast. Med. Eng. Phys..

[43] D’Orsi C.J., Sickles E.A., Mendelson E.B., Morris E.A. (2013). ACR BI-RADS Atlas: Breast Imaging Reporting and Data System.

[44] Hansen J., Netter F. (2014). Netter’s Clinical Anatomy.

[45] Clough K.B., Kaufman G.J., Nos C., Buccimazza I., Sarfati I.M. (2010). Improving Breast Cancer Surgery: A Classification and Quadrant per Quadrant Atlas for Oncoplastic Surgery. Ann. Surg. Oncol..

[46] Tsai C.F., Wu J.W. (2008). Using neural network ensembles for bankruptcy prediction and credit scoring. Expert Syst. Appl..

[47] Ho T.K. (2002). A Data Complexity Analysis of Comparative Advantages of Decision Forest Constructors. Pattern Anal. Appl..

[48] Breiman L. (1999). Prediction Games and Arcing Algorithms. Neural Comput..

[49] Mason L., Baxter J., Bartlett P., Frean M. (1999). Boosting algorithms as gradient descent. Proceedings of the 12th International Conference on Neural Information Processing Systems.

[50] Chen T., Guestrin C. (2016). XGBoost: A Scalable Tree Boosting System. CoRR.

[51] Friedman J.H. (2001). Greedy function approximation: A gradient boosting machine. Ann. Stat..

[52] Borchani H., Varando G., Bielza C., Larrañaga P. (2015). A survey on multi-output regression. Wiley Interdiscip. Rev. Data Min. Knowl. Discov..

[53] Pedregosa F., Varoquaux G., Gramfort A., Michel V., Thirion B., Grisel O., Blondel M., Prettenhofer P., Weiss R., Dubourg V. (2011). Scikit-learn: Machine Learning in Python. J. Mach. Learn. Res..

[54] Liaw A., Wiener M. (2002). Classification and Regression by randomForest. R News.

[55] Chan J.C.W., Paelinckx D. (2008). Evaluation of Random Forest and Adaboost tree-based ensemble classification and spectral band selection for ecotope mapping using airborne hyperspectral imagery. Remote Sens. Environ..

